# Liver mitochondria-associated endoplasmic reticulum membrane proteomics for studying the effects of ZiBuPiYin recipe on Zucker diabetic fatty rats after chronic psychological stress

**DOI:** 10.3389/fcell.2022.995732

**Published:** 2022-11-03

**Authors:** Huiying Xu, Wen Zhou, Libin Zhan, Tingting Bi, Xiaoguang Lu

**Affiliations:** ^1^ Modern Research Laboratory of Spleen Visceral Manifestations Theory, School of Traditional Chinese Medicine, School of Integrated Chinese and Western Medicine, Nanjing University of Chinese Medicine, Nanjing, China; ^2^ Center for Innovative Engineering Technology in Traditional Chinese Medicine, Liaoning University of Traditional Chinese Medicine, Shenyang, China; ^3^ Key Laboratory of Ministry of Education for TCM Viscera-State Theory and Applications, Liaoning University of Traditional Chinese Medicine, Shenyang, China; ^4^ Department of Emergency Medicine, Zhongshan Hospital, Dalian University, Dalian, China

**Keywords:** chronic psychological stress, type 2 diabetes mellitus, ZiBuPiYin recipe, mitochondria-associated ER membrane, proteomics

## Abstract

Type 2 diabetes mellitus (T2DM) is a complex metabolic disease with multiple etiologies, involving both genetic and environmental factors. With changes associated with modern life, increasing attention has been paid to chronic psychological stressors such as work stress. Chronic psychological stress can induce or aggravate diabetes mellitus, and conversely, with the deterioration of T2DM, patients often experience different degrees of depression, anxiety, and other negative emotions. In order to clarify the role of ZiBuPiYin recipe (ZBPYR) in regulating the liver mitochondria-associated endoplasmic reticulum membrane proteome to improve T2DM with chronic psychological stress, differentially expressed proteins (DEPs) were identified among Zucker lean littermates (control group), chronic psychological stress T2DM rats (model group), and ZBPYR administration rats (ZBPYR group) through iTRAQ with LC-MS/MS. Using Mfuzz soft clustering analysis, DEPs were divided into six different clusters. Clusters 1–6 contained 5, 68, 44, 57, 28, and 32 DEPs, respectively. Given that ZBPYR can alleviate T2DM symptoms and affect exploratory behavior during T2DM with chronic psychological stress, we focused on the clusters with opposite expression trends between model:control and ZBPYR:model groups. We screened out the DEPs in clusters 1, 3, and 4, which may be good candidates for the prevention and treatment of T2DM with chronic psychological stress, and further conducted bioinformatics analyses. DEPs were mainly involved in the insulin signaling pathway, oxidative phosphorylation, tricarboxylic acid cycle, amino acid metabolism, lysosome-related processes, and lipid metabolism. This may indicate the pathogenic basis of T2DM with chronic psychological stress and the potential therapeutic mechanism of ZBPYR. In addition, two key proteins, lysosome-associated protein (Lamp2) and tricarboxylic acid cycle-related protein (Suclg1), may represent novel biomarkers for T2DM with chronic psychological stress and drug targets of ZBPYR. Western blot analyses also showed similar expression patterns of these two proteins in liver MAMs of the model and ZBPYR groups.

## Introduction

Type 2 diabetes mellitus (T2DM) is a metabolic disease caused by the loss of insulin sensitivity and pancreatic *β*-Cells failure. The basic characteristic of T2DM is hyperglycemia, and typical symptoms of T2DM are polydipsia, polyuria, polyphagia, and weight loss. The pathogenesis of T2DM is complicated and still not completely understood. A group of emerging literatures show that stress plays an important role in the etiology of T2DM, which is not only a predictor of new onset T2DM, but also a prognostic factor for patients with T2DM (Sui et al., 2016; Hackett and Steptoe, 2017). Repeated or continuous stress exposure results in chronic unsteady load, accompanied by glucose metabolism disorder and neuroendocrine dysfunction, as well as chronic low-grade inflammation. In patients with confirmed diabetes mellitus, depression and diabetes-related distress are associated with poor glycemic control and cardiovascular complications (Hackett and Steptoe, 2017). Therefore, the chronic psychological stress T2DM model has been established by chronic psychological stress stimulation in well-accepted Zucker diabetic fatty (ZDF) rats with T2DM. We used this model to elucidate the specific molecular mechanisms underlying T2DM with chronic psychological stress.

The liver, as one of the three main extra-pancreatic insulin-sensitive organs, plays an essential role in coordinating systemic metabolic homeostasis and adapting to nutrient supply and deficiency. In a physiological state, the combined action of glucagon and insulin allows the precise regulation of hepatic glucose output (Galicia-Garcia et al., 2020). During insulin-resistant states such as T2DM, the physiological level of circulating insulin is insufficient to trigger an appropriate insulin response in hepatocytes (Meshkani and Adeli, 2009). In the liver, insulin resistance fails to inhibit glucose production, enhances lipogenesis, impairs glycogen synthesis, and increases the synthesis of proteins such as the pro-inflammatory C-reactive protein (Galicia-Garcia et al., 2020). Therefore, it is necessary to study the changes of liver function in T2DM with chronic psychological stress.

Mitochondria-associated endoplasmic reticulum membrane (MAM) is a special domain that mediates the tight junctions between mitochondria and the endoplasmic reticulum (ER), and it constitutes the physical basis for communication between two organelles. It is known that these associations play an significant role in maintaining intracellular homeostasis. Impaired MAM signaling has broad implications in a great deal of diseases, such as diabetes mellitus and obesity. Growing evidence suggests that MAMs affect insulin signaling through different pathways, including those related to mitochondrial function, ER stress responses, calcium signaling, lipid metabolism, and inflammation (Cheng et al., 2020). Continuing proteomics researches have demonstrated changes of MAM proteins in different tissues (e.g., liver, testis, retina, brain) in mice or humans (Sala-Vila et al., 2016; Ma et al., 2017a; Wang et al., 2018b; Tubbs et al., 2018). In the liver, MAMs are key glucose-sensing regulators and considered to be a hub for insulin signaling (Cheng et al., 2020). Therefore, A comprehensive understanding of the protein composition of hepatic MAMs will be especially useful for clarifying the mechanisms of T2DM with chronic psychological stress.

The ZiBuPiYin recipe (ZBPYR) is modified on the basis of the Zicheng Decoction, which was recorded in “Bujuji” written by Cheng Wu in the Qing dynasty. The research of our team have indicated that ZBPYR improves diabetic symptoms and central complications in diabetic model rats. Its mechanism may be related to the regulation of autophagy, ER stress, insulin signaling, insulin-degrading enzyme expression, and gut microbiota (Liang et al., 2013; Liang et al., 2015; Gu et al., 2017). Therefore, it is of great significance to explore the underlying mechanisms connecting the liver MAM proteome to T2DM with chronic psychological stress and ZBPYR treatment using isobaric tags for relative and absolute quantitation (iTRAQ) with liquid chemistry-mass spectrometry/mass spectrometry (LC-MS/MS) technology.

## Materials and methods

### Animals and treatments

Six-week-old male ZDF (fa/fa) rats and their Zucker lean (ZL, fa/+) littermates of the same age were both purchased from Vital River Laboratories (Beijing, China). During the experiment, the rats were placed in the specific, pathogen-free animal experiment center in Nanjing University of Chinese Medicine, and exposed to relative humidity (65% ± 5%), controlled temperature (24°C ± 2°C) and artificial light (12 h light/dark cycle) conditions. They had access to water (autoclaved before use) *ad libitum* with a standard diet for ZL rats and Purina 5008 chow for ZDF rats. Food and water intake and body weights were measured daily.

After 1 week of adaptive feeding, ZDF rats were randomized into two groups: chronic psychological stress T2DM rats (model group, *n* = 6) and psychological stress combined with ZBPYR administration rats (ZBPYR group, *n* = 6). They were all subjected to 6 weeks of stressful stimulation, which included restriction, rotation, and congestion. The stress procedures were performed as previously described in detail (Bi et al., 2020). Additionally, ZL rats were used as a control group (*n* = 6). All animal work was approved by the Animal Ethics Committee of Nanjing University of Chinese Medicine (Approval No. ACU170606) and performed according to the National Institutes of Health Guide for the Care and Use of Laboratory Animals.

### Random blood glucose test, insulin tolerance test, and oral glucose tolerance test

From weeks 7 to 15, the Random blood glucose (RBG) levels were measured. In the insulin tolerance test (ITT), all rats’ blood samples were collected at 0, 15, 30, 60, 90, and 120 min after the injection of insulin (0.5 U/kg body weight) by fasting for 6 h. And in the oral glucose tolerance test (OGTT), blood samples were collected at 0, 30, 60, 90, and 120 min after the administration of glucose (2 g/kg body weight) by fasting for 14 h. Blood glucose values were measured by tail blood.

### Open field test

Before euthanasia, the rats were exposed to the open field test (OFT). Briefly, they were placed in the test environment for 1 h before test. During the test, they were allowed to explore freely in a dimly-lit OFT chamber (50 cm × 50 cm × 50 cm) for 5 min. The square site was divided into a center area (10 cm × 10 cm) and surrounding area. The patterns of behavior measured in the OFT included total distance, number of center entries, time spent in center, vertical numbers, defecation numbers, and episodes of grooming. At test intervals, the OFT chamber was cleaned with 75% ethanol and dried to remove interference.

### Tissue collection and mitochondria-associated endoplasmic reticulum membrane isolation

All rats (*n* = 18) were decapitated after ether anesthesia. The liver samples were dissected on ice and weighed immediately.

After a little modification, we isolated liver MAM according to an established protocol (Wieckowski et al., 2009). Briefly, liver tissues were manually homogenized, and nuclei and unbroken cells were pelleted by centrifugation. The collected supernatant was then centrifuged to separate the crude mitochondria. After several washes, it was suspended in 4 ml resuspending buffer III, which was layered on top of 16 ml of 30% Percoll medium. And all of them were centrifuged at 95,000 × g for 30 min. The MAM fraction and pure mitochondrial fraction were separately extracted from the Percoll gradient and further purified by centrifugation. The above operations were completed on ice or at 4°C. All fractions were rapidly frozen with liquid nitrogen and stored at −80°C before use.

### Digestion of liver mitochondria-associated endoplasmic reticulum membrane samples and isobaric tags for relative and absolute quantitation labeling

100 μl sample was digested in parallel by filter-aided sample preparation. The filter-aided sample preparation method was as follows. Firstly, 200 μl UA buffer (8 M urea and 150 mM Tris-HCl, pH 8.0) were added to each sample. Secondly, DTT was added to form a mixed sample with a final concentration of 100 mM, which was left at room temperature for 1.5 h. Thirdly, each mixture was moved to an ultrafiltration filter (30 kDa cutoff, Sartorius, Germany) and centrifuged at 13,000 × g for 20 min; repeat this process twice and then discard the filtrate. In addition, the filtered sample was added to 100 μl iodoacetamide solution (50 mM iodoacetamide in UA buffer), shaken at 600 rpm for 1 min, kept away from light at room temperature for 30 min, and centrifuged at 13,000 × g for 20 min. Subsequently, 100 μl UA buffer was added to the filter unit and centrifuged at 13,000 × g for 20 min; this process was repeated twice. Moreover, 100 μl NH_4_HCO_3_ buffer (Sigma, St. Louis, MO) was added to the filtered sample and centrifuged at 13,000 × g for 15 min; repeat this step three times. Then, 40 μl of trypsin buffer (3 μg trypsin in 40 μl NH_4_HCO_3_ buffer) was added to each sample, shaken at 600 rpm for 1 min, and digested at 37°C for 16 h–18 h. The filtered sample was transferred to a new tube and centrifuged at 13,000 × g for 30 min. Finally, 40 μl NH_4_HCO_3_ buffer was added and centrifuged at 13,000 × g for 30 min. The resulting peptides were collected and desalted with a C18-SD Extraction Disk Cartridge (66872-U Sigma). The peptide concentration was analyzed by OD280.

Most improtant of all, 50 μg of peptide sample were labeled with iTRAQ reagents according to the manufacturer’s instructions (iTRAQ Reagent-8plex Multiplex Kit, Applied Biosystems SCIEX, Foster City, CA). The liver MAM samples of the control, model, and ZBPYR groups, as well as IS samle were all labeled with labeling reagents. Among them, the IS sample was a mixed sample made from the same mass of protein mixtures from different groups. Then, three independent biological experiments were performed for triplicate LC-MS/MS analyses.

### LC-MS/MS analysis

The combination of EASY-nLC 1000 system (Thermo Fisher Scientific, Odense, Denmark) and Q-Exactive (Thermo Finnigan, San Jose, CA) mass spectrometer was performed. Peptide mixtures were loaded into the Thermo Scientific EASY column (2 cm × 100 μm, 5 μm-C18), then separated with the Thermo Scientific EASY column (75 μm × 100 mm, 3 μm-C18). The mobile phase consisted of water (A, containing 0.1% formic acid)/acetonitrile aqueous solution (B, containing 0.1% formic acid; acetonitrile 84%) and was delivered at a flow rate of 250 nl/min. The segmented gradient program was as follows: B gradient elution at 0%–55% between 0 and 220 min, 55%–100% between 220 and 228 min, and 100% between 228 and 240 min.

The Q-Exactive mass spectrometer was set to perform data acquisition in positive ion mode with a selected mass range of 350–1,800 mass/charge (m/z). The resolving power was set as 70,000 for the MS scan and 17,500 for the MS/MS scan at m/z 200. The maximum ion injection times was 20 ms for the survey scan and 60 ms for the MS/MS scans. And the automatic gain control target values for the MS scan mode was set to 3e6. Dynamic exclusion for selected precursor ions was set at 30 s. MS/MS data were acquired using the top 10 most abundant precursor ions with charge ≥2 as determined from the MS scan. These were selected with an isolation window of 2 m/z and fragmented with normalized collision energies of 29 eV. The underfill ratio was defined as 0.1%.

### Database searching and protein quantification

The raw files were searched using the MASCOT engine (Matrix Science, London, UK; v2.2) and Proteome Discoverer 1.4, against the Uniprot Rat database (02-28-2015, 34,164 entries). The following settings were selected: Enzyme: Trypsin; Fixed modifications: Carbamidomethyl (C), iTRAQ8plex (N-term), iTRAQ8plex (K); Variable modifications: Oxidation (M), iTRAQ8plex (Y); Peptide mass tolerance: ± 20 ppm; Fragment mass tolerance: 0.1 Da. Only proteins identified at peptide FDR ≤1% were considered for further downstream analysis. To determine differentially expressed proteins (DEPs), it must be identified and quantified with at least one significant peptide and the *p*-values of protein quantitation in the control, model, and ZBPYR groups should be less than 0.05. In order to further determine the key proteins, three screening criteria need to be met, which are as follows: 1) the fold change of protein relative abundance >±1.2; 2) *t*-test *p*-value <0.05; and 3) the opposite expression trend of upregulation and downregulation between model:control and ZBPYR:model groups.

### Bioinformatics analysis

For global analysis, 806 proteins analyzed in triplicate experiments were used. Through the DAVID bioinformatics resource (v6.8) (https://david.ncifcrf.gov/), we performd gene ontology (GO) (http://geneontology.org/) annotation and Kyoto Encyclopedia of Genes and Genomes (KEGG) pathway enrichment analysis. Soft clustering analysis was performed using Mfuzz to mine the expression patterns of 234 DEPs in the control, model, and ZBPYR groups. Using a self-written Perl script, GO, KEGG pathway, and protein domain enrichment analyses were achieved, in which all the rat proteins were used as the basis to calculate enrichment values. The significance of the enrichment values was tested, which were then represented in a heatmap. To search for protein-protein interaction (PPI) networks of the DEPs, STRING database version 11.0 (https://string-db.org/) was used. The PPI network was visualized using Cytoscape version 3.7.1. Functional modules in the PPI network were identified using the MCODE plugin of Cytoscape with default parameters. ImageGP (v1.0) was used to generate bubble chart and volcano plot.

### Western blot analysis

Samples were lysed in a radio immunoprecipitation assay buffer (Beyotime, China) with a protease and phosphatase inhibitor cocktail (Cell Signaling Technology, United States). The protein samples were measured by a BCA protein assay kit (Beyotime, China). Fifty micrograms of liver MAM protein were separated by SDS-PAGE and transferred to a polyvinylidene difluoride membrane. After blocking with 5% skim milk in Tris-buffered saline containing 0.05% Tween-20, membranes were incubated with appropriate primary antibodies overnight at 4°C and then incubated with the HRP-conjugated secondary antibody for 1.5 h at room temperature. The primary antibodies were listed in Supplementary Table S1. The membranes were developed with an ECL kit (Tanon, China) using an Amersham Imager 600 (General Electric Company, United States).

### Statistical analysis

All the data were expressed as the mean ± standard error of measurement (SEM). Data were statistically analyzed by Perseus software (version 1.6.14.0) (http://www.perseus-framework.org) and GraphPad Prism 6.0 statistical software (GraphPad Software, United States). Comparisons among multiple groups were performed by one-way analysis of variance followed by a post hoc Turkey’s test. Statistical significance was defined as *p* < 0.05.

## Results

### ZiBuPiYin recipe administration attenuated type 2 diabetes mellitus symptoms in Zucker diabetic fatty rats

**FIGURE 1:**
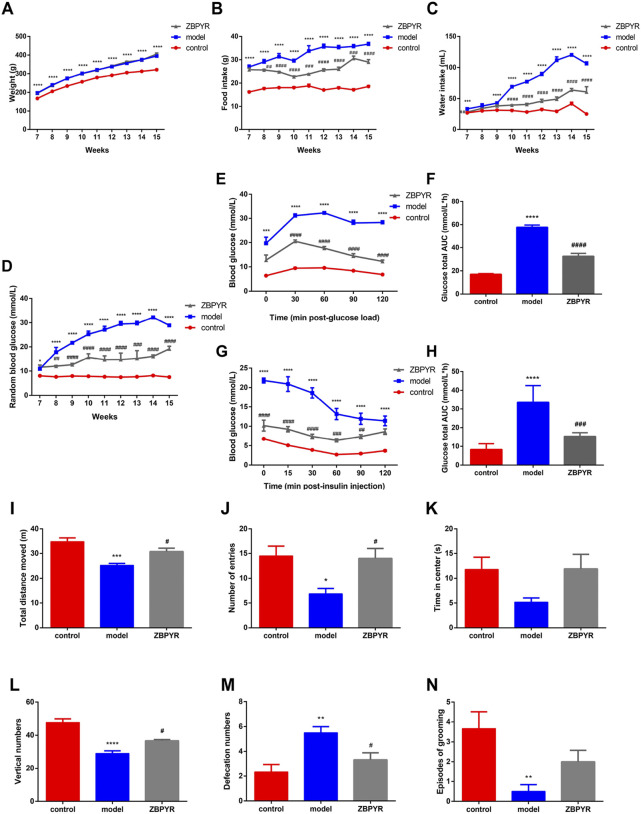
Effects of ZBPYR on physiological parameters, RBG, OGTT, ITT, and exploratory behavior. **(A–C)** Effects of ZBPYR on body weight **(A)**, food intake **(B)**, and water intake **(C)**. These physiological paramete were measured daily at 7–15 weeks old. **(D–H)** Effects of ZBPYR on RBG, OGTT, and ITT. RBG **(D)** was measured at 7–15 weeks of age. Line charts show the levels of blood glucose in the OGTT **(E)** and ITT **(G)**, respectively. Histograms show the total glucose area under the curve (AUC) in the OGTT **(F)** and ITT **(H)**, respectively. **(I–N)** Effect of ZBPYR on activities in the open-field test. Total distance **(I)**, number of entries **(J)**, time in center **(K)**, vertical numbers **(L)**, defecation numbers **(M)**, and episodes of grooming **(N)** were measured. *p < 0.05, **p < 0.01, ***p < 0.001, ****p < 0.0001, model vs. control; #*p* < 0.05, ##*p* < 0.01, ###*p* < 0.001, ####*p* < 0.0001, ZBPYR vs. model (mean ± SEM, *n* = 6 per group).

With increasing age, the body weights were continuously elevated in the model and ZBPYR groups. Specifically, the body weights of the model group increased by 22.62%, 14.00%, 9.73%, 8.68%, 4.24%, 4.80%, 2.35%, and 2.79% each week during 8–15 weeks, while those of the ZBPYR group increased by 21.81%, 14.49%, 9.81%, 6.31%, 5.54%, 5.43%, 5.19%, and 5.31%, respectively. The weight of the control group was significantly lower than that of model and ZBPYR groups of the same age (Figure 1A). With the increase of age, the food and water intake of model and ZBPYR groups increased variably, but those of control group were relatively stable (Figures 1B,C). At weeks 7–15, the food intake of the control group was consistently less than that of the model group, while the food intake of the ZBPYR group was significantly less than that of model group at weeks 8–15. In addition, at weeks 7 and 9–15, the water intake of the model group was significantly more than that of the control group. And at week 7 and 10–15, the water intake of the ZBPYR group was significantly less than that of the model group. In summary, T2DM with chronic psychological stress significantly increased body weight, food intake, and water consumption, while ZBPYR reduced the increase of food and water consumption, but had little effect on body weight.

### ZiBuPiYin recipe administration ameliorated glucose metabolism in Zucker diabetic fatty rats

As age increased, the blood glucose of the model group increased rapidly, while that of the ZBPYR group increased slowly, and that of the control group was relatively stable (Figure 1D). The levels of RBG were weekly increased by 63.05%, 21.82%, 16.37%, 7.40%, 8.67%, 0.68%, and 8.66% in the model group during weeks 8–15, and increased by 3.60%, 5.28%, 23.88%, −5.86%, 0.23%, 3.05%, and 5.70% in the ZBPYR group. Within the OGTT and the ITT, the fasting blood glucose levels of the control group were lower than those of the model group, which in turn were higher than those of the ZBPYR group (Figures 1E–H). At the last time point, the blood glucose in the control and ZBPYR groups almost recovered to the value of 0 min in the OGTT, while those of the two group were slightly lower than the initial level of 0 min in the ITT. In conclusion, T2DM with chronic psychological stress significantly increased blood glucose and decreased insulin sensitivity, while ZBPYR administration significantly decreased blood glucose and increased insulin sensitivity.

### ZiBuPiYin recipe regulated the general state and exploratory behavior of Zucker diabetic fatty rats

Both the model and ZBPYR groups were subjected to three types of stressful stimulus: restriction, rotation, and congestion, for 6 weeks. The control rats were in a good mental state and had flexible reactions during the whole stress process. In the initial stage of the stress, the model group showed strong resistance, hissing, and refusal to enter the operation box. During the modeling period, the amount of feces and urine increased, and the respiratory rate accelerated. In the late stage of the stress experiment, the model group showed weakened resistance, slow reaction and activity, mental depression, and scattered and dull hair. The ZBPYR group was similar to the model group in the early stage of stress experiment, but was more active in the late stage of the stress experiment and had moderate response to external stimuli.

The OFT is a method for assessing the autonomic and exploratory behavior, and tension of experimental animal in a novel environment. The frequency and duration of exploration behavior of laboratory animals reflect their exploratory behavior, the frequency of urine and defecation reflects their tension, and the frequency of grooming reflects their alertness and self-concern. Compared with the control group, the total distance of movement, the frequency of entering the center, and vertical numbers were significantly decreased in the model group (Figures 1I,J,L), and the time spent in the center had a decreasing trend (Figure 1K). After ZBPYR administration, the exploratory behavior of rats in the ZBPYR group returned to normal. Compared with the control group, the defecation volume of the model group was significantly increased, but decreased after ZBPYR administration (Figure 1M). The amount of grooming in the model group was significantly reduced compared with the control group, and had a increasing trend after ZBPYR administration (Figure 1N). In short, the rats in the model group showed reduced exploratory behaviors and might have had anxiety, but ZBPYR administration improved thier behavior.

### Protein identification and quantification

**FIGURE 2:**
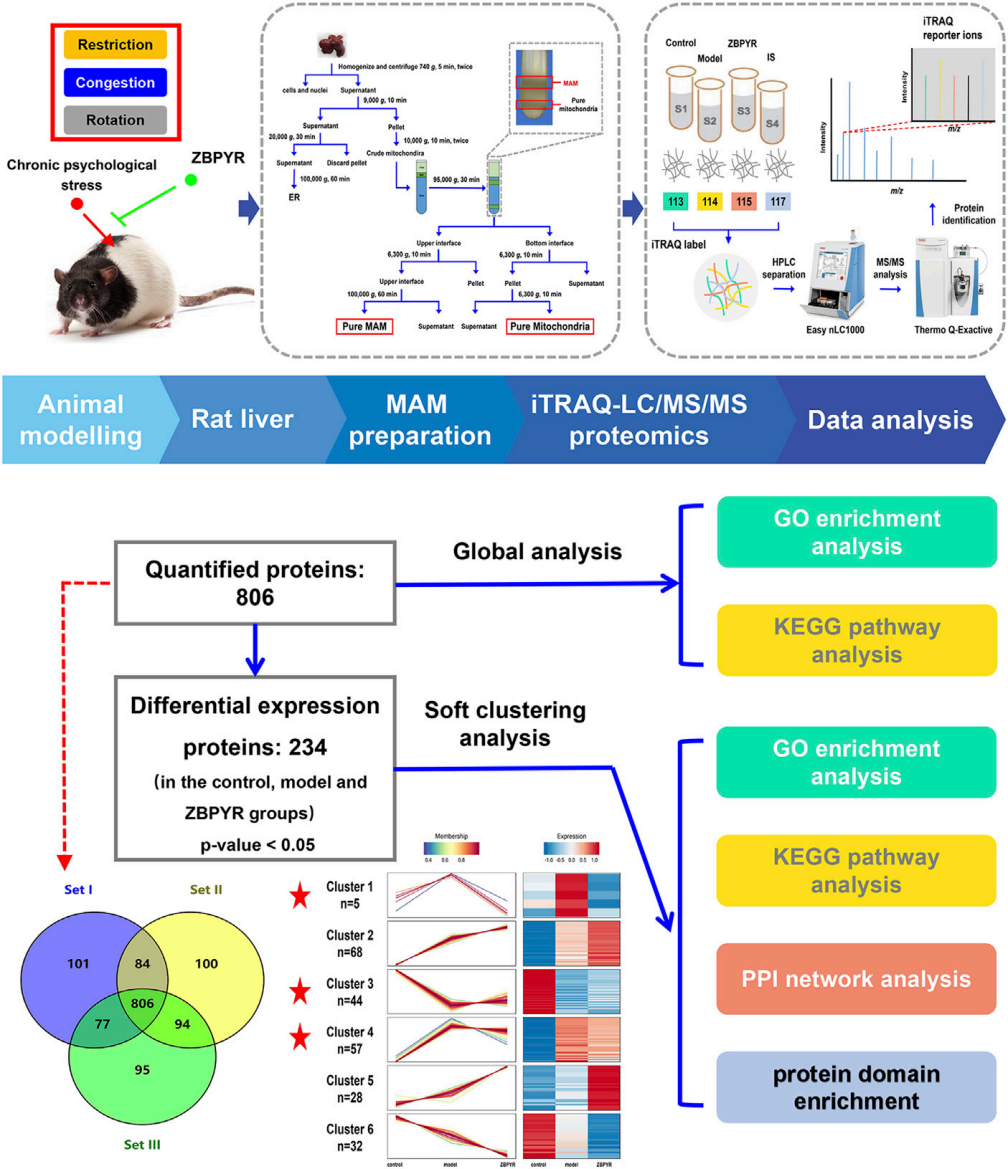
Proteomics workflow. The animal was exposure to three chronic psychological stressors (i.e., restriction, rotation, and congestion). Fresh liver tissue MAM was extracted using gradient centrifugation, and lysis proteins were labeled by an iTRAQ 8-plex. The peptides were subsequently analyzed by LC-MS/MS technology. Peptides were identified and quantified according to the iTRAQ reporter area, and relative protein quantification was inferred from these values. The 234 differentially expressed proteins (DEPs) of the control, model, and ZBPYR groups were analyzed by global analysis and soft clustering analysis. Based on bioinformatics analysis (GO enrichment analysis, KEGG pathway analysis, PPI network analysis, and domain enrichment analysis), key pathways and proteins were screened.

**FIGURE 3:**
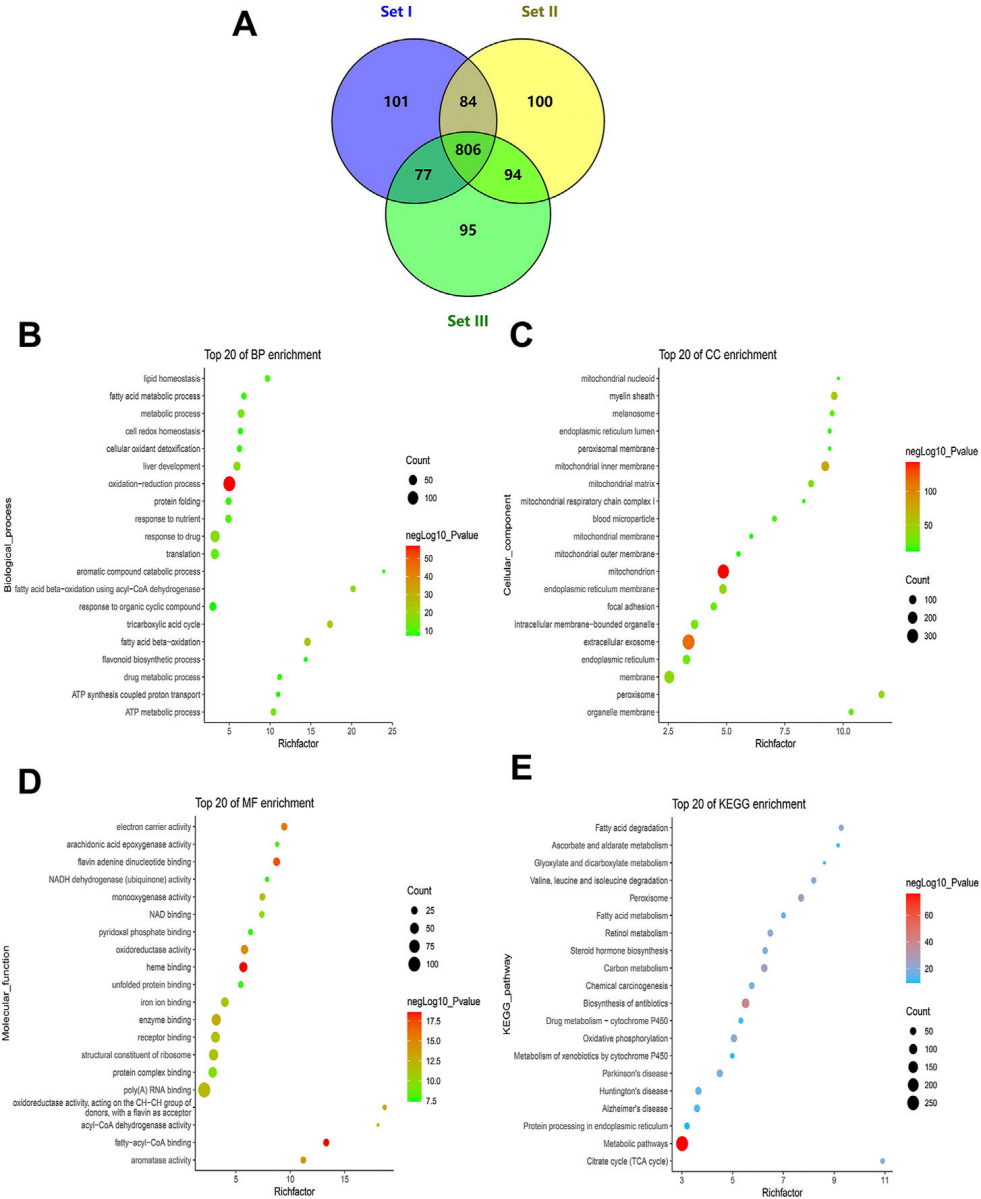
Profile of quantitative MAM proteins identified in the liver. **(A)** Venn diagram of the number of non-redundant proteins in technical triplicate analysis. **(B–D)** Profile of GO enrichment analysis of the quantitative proteins, including biological process (BP), cellular component (CC), and molecular function (MF). Bubble charts show the terms of BP **(B)**, CC **(C)**, and MF **(D)**, respectively. **(E)** A bubble chart showing terms of the KEGG pathway. The top 20 terms sorted by *p*-values for each category are displayed, with the *y*-axis showing the categories (BP, CC, MF, and KEGG) and the *x*-axis showing the rich factor (rich factor = number of DEPs enriched in the categories/number of all proteins in background protein set). The color and size of bubbles indicate the significance of enrichment and the number of DEPs enriched in the categories, respectively.

The workflow for proteomic analysis is shown in Figure 2. To start with, liver MAM lysates of the control, model, and ZBPYR groups were prepared in triplicate. The iTRAQ labeling-based proteomic technology was performed to quantify the relative abundance of proteins in MAM sample. Next, the peptide mixtures were analyzed by MS/MS on a Q-Exactive mass spectrometer coupled with the EASY-nLC1000 system. Subsequently, raw data produced by the mass spectrometric analysis were searched using the MASCOT server 2.2 against the UniProt-rat database, which yielded 1,357 proteins from rat liver MAM. For more reliable unique protein identification, we screened the proteins observed in all three biological replicates for further study. A total of 806 quantitative proteins from rat liver MAM were identified in the iTRAQ experiments (Figure 3A; Supplementary Table S2). Among them, in order to verify the quality of rat liver MAM fractions, we performed quality control on different steps of MAM preparation of ZL rat livers (*n* = 3) by western blot analysis (Supplementary Figure S1). The distribution trend of six molecules, such as MAM labeled acyl-CoA synthase long chain 4 (ACSL4/FACL4) in different fractions was consistent with the previously published data (Sonn et al., 2021; Xue et al., 2021).

### Quantitative proteomic analysis of liver mitochondria-associated endoplasmic reticulum membrane proteins

The DAVID online database was used to conduct enrichment analysis of 806 quantitative proteins. GO analysis is divided into three parts: biological process (BP), cellular component (CC), and molecular function (MF). In BP, the top 20 significantly enriched entries included the oxidation-reduction process, ATP metabolic process, tricarboxylic acid cycle, fatty acid beta-oxidation, protein folding, lipid homeostasis, liver development, and drug metabolic process (Figure 3B). In CC, the top significant terms were mitochondria (i.e., mitochondrial inner membrane, mitochondrial outer membrane, mitochondrial matrix, mitochondrial respiratory chain complex I, and mitochondrial nucleoid), ER (i.e., ER membrane and ER lumen), intracellular membrane-bounded organelles, extracellular exosomes, the myelin sheath, and peroxisomes (Figure 3C). In MF, electron carrier activity, oxidoreductase activity, NAD binding, NADH dehydrogenase (ubiquinone) activity, flavin adenine dinucleotide binding, unfolded protein binding, fatty-acyl-CoA binding, acyl-CoA dehydrogenase activity, arachidonic acid epoxygenase activity, heme binding, and iron ion binding were among the top 20 significantly enriched terms (Figure 3D). The KEGG pathway analysis revealed some important pathways such as carbon metabolism; oxidative phosphorylation; fatty acid metabolism; the citrate cycle; valine, leucine and isoleucine degradation; retinol metabolism; Alzheimer’s disease; Parkinson’s disease; Huntington’s disease; Steroid hormone biosynthesis; protein processing in the ER; and drug metabolism-cytochrome P450 (Figure 3E). It is worth noting that KEGG analysis revealed some pathways related to metabolic pathways and “protein processing in ER,” which is likely because the attribution of MAM dysfunction to metabolic diseases (such as T2DM) has been attracting attention, and much reliable evidence has proved this point. Taken together, the liver MAM quantitative proteins were mainly related to mitochondria, ER, and liver-major functions.

### Soft clustering of protein expression patterns among the control, model, and ZiBuPiYin recipe groups

To identify differentially regulated proteins, One-way analysis of variance was used to analyze the significance among the control, model, and ZBPYR groups. Proteins with *p* < 0.05 were screened as DEPs. Mfuzz is used for Soft clustering to mine the expression patterns of differential proteins in the three groups.

Soft clustering analysis was performed to compare the expression ratios of 234 DEPs from three groups. According to the different expression profiles among the groups, the proteins were divided into six different clusters (Supplementary Figure S2; Supplementary Table S3). Clusters 1–6 included 5, 68, 44, 57, 28, and 32 DEPs, respectively. Since ZBPYR can alleviate T2DM symptoms and improve the exploratory behavior caused by T2DM with chronic psychological stress, we focused on clusters with opposite expression trends between model:control and ZBPYR:model groups. In total, 62 proteins fell into clusters 1 and 4. These clusters included proteins whose expressions were significantly increased in the model group compared with the control group and were suppressed in the ZBPYR group. Apart from clusters 1 and 4, the expressions of most proteins in cluster 3 (44 DEPs) were reduced in the model group compared to the control group, but their expression was restored by ZBPYR treatment. These results suggest that the proteins in clusters 1, 3, and 4 are candidates for the prevention or treatment of T2DM with chronic psychological stress.

### Gene ontology enrichment analysis of differentially expressed proteins in liver mitochondria-associated endoplasmic reticulum membrane based on soft clustering

**FIGURE 4:**
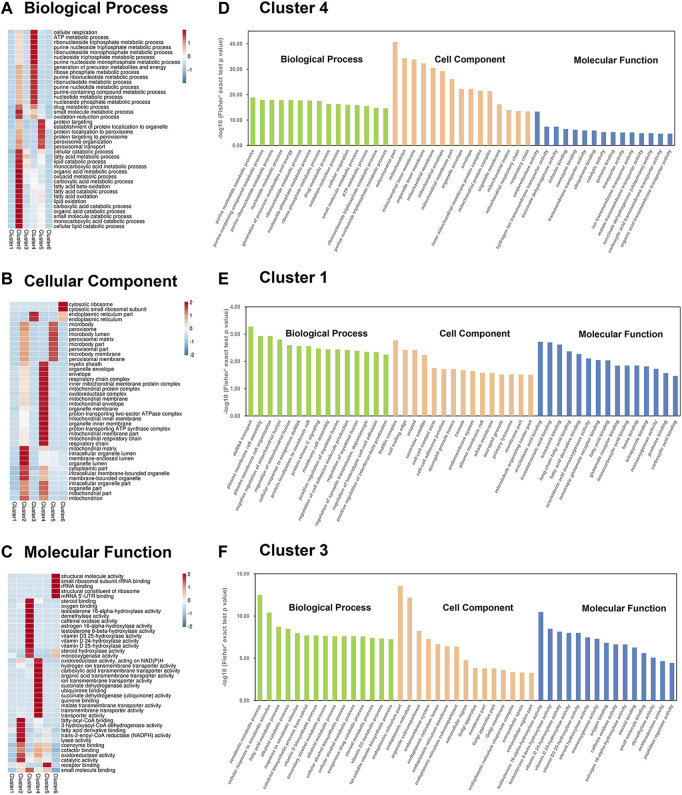
Enrichment based on Mfuzz clustering by three GO annotations. **(A–C)** Three GO annotated heatmaps of proteins were obtained: **(A)** BP, **(B)** CC, and **(C)** MF, with altered expression levels among the six Mfuzz clustering groups. The heatmap scale is based on *Z* scores ranging from −2/−1 (blue) to 1/2 (red), with a midpoint of 0 (white). **(D–F)** GO-based enrichment analysis is divided into cluster 1 **(D)**, cluster 4 **(E)**, and cluster 3 **(F)**. The BP, CC, and MF are labeled in green, orange, and blue, respectively.

GO enrichment analysis showed that the six clusters were largely related to specific BP, MF, and CC according to the heatmap (Figures 4A–C; Supplementary Table S4). In cluster 4, proteins involved in mitochondrial functions (i.e., respiratory chain complex, mitochondrial protein complex, oxidoreductase complex, and mitochondrial respiratory chain) formed a specific focus in the GOCC. In particular, cluster 4 included proteins related to ATP metabolic processes and nucleotide metabolic processes (i.e., nucleoside triphosphate metabolic and purine nucleoside monophosphate metabolic processes) in GOBP and proteins related to oxidoreductase activity and transmembrane transporter activity (i.e., transmembrane transporter activity of hydrogen ions, and carboxylic acid, organic acid, and malate transport) in GOMF. In the histogram containing the top 15 items sorted by p value, strong enrichment of drug metabolic process and small molecule metabolic process were found in cluster 4 (Figure 4D). A specific focus of GO terms in cluster 1 was not observed in the heat map, so the top 15 terms sorted by *p*-value was displayed in the histogram (Figure 4E). In cluster 1, GOBP demonstrated strong enrichment of plasma membrane raft assembly and organization, the regulation of mitochondrial fusion, and protein kinase C signaling, and GOCC included proteins related to the cell-cell contact zone, endomembrane system, plasma membrane rafts, and primary lysosomes. GOMF enrichment demonstrated that most of the DEPs were involved in binding, including arachidonic acid binding, fatty acid binding, glutamate receptor binding, protease binding, and carboxylic acid binding. The protein expression trend of DEPs in cluster 3 was opposite to that in clusters 1 and 4. Cluster 3 contained the ER proteins enriched in GOCC and proteins related to lipid catabolic processes in GOBP. Liver-specific functional proteins such as those for steroid binding, sex hormone biosynthesis (i.e., testosterone 16-alpha-hydroxylase activity, testosterone 6-beta-hydroxylase activity, and estrogen 16-alpha-hydroxylase activity), and vitamin D metabolism (i.e., vitamin D 25-hydroxylase activity, vitamin D3 25-hydroxylase activity, and vitamin D 24-hydroxylase activity) were enriched in cluster 3 GOMF. Additionally, we also observed strong enrichment of vitamin D biosynthetic and cellular alcohol metabolic processes in the histogram (Figure 4F).

### Kyoto encyclopedia of genes and genomes enrichment analysis of differentially expressed proteins in liver mitochondria-associated endoplasmic reticulum membrane based on soft clustering

**FIGURE 5:**
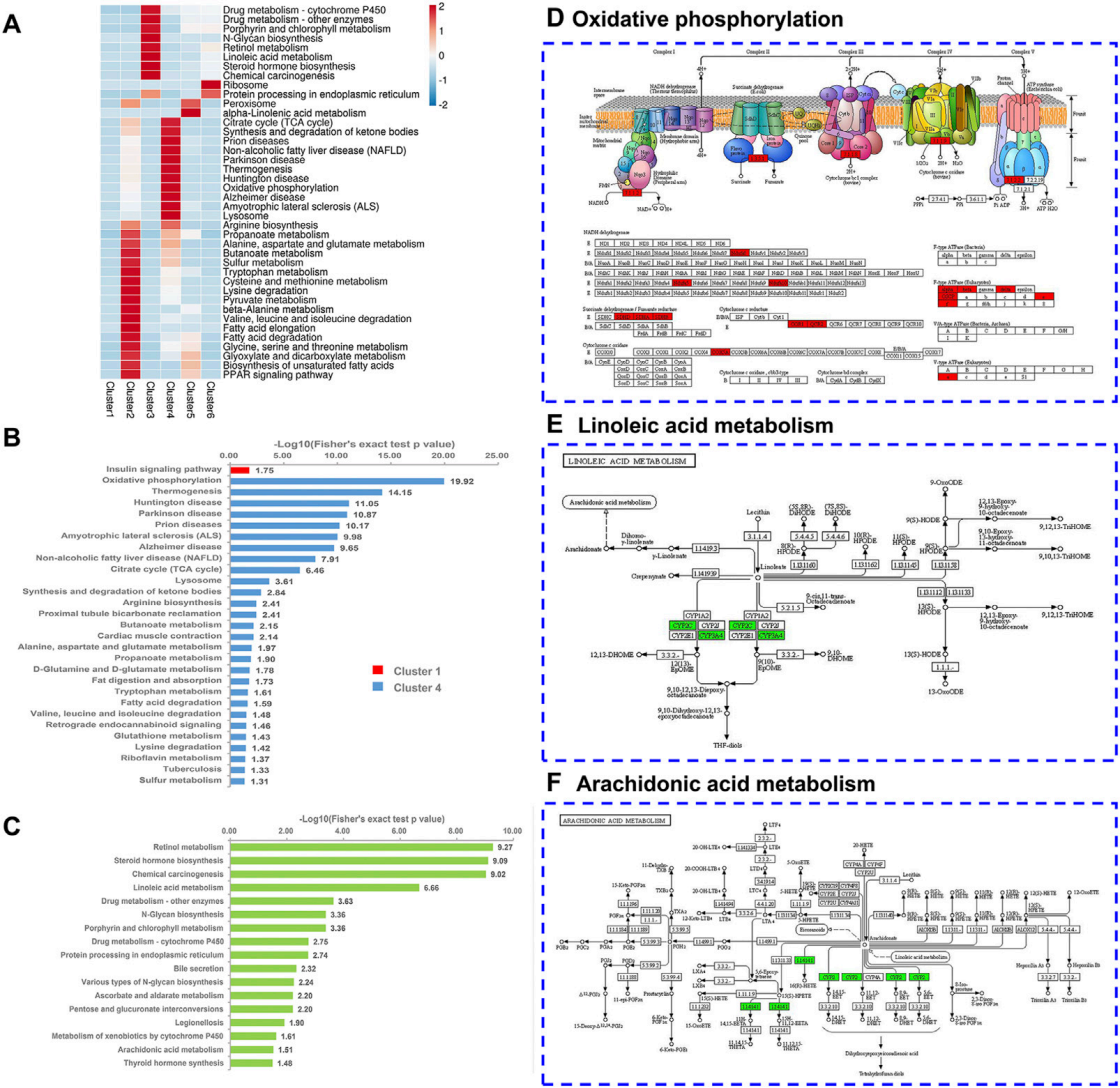
KEGG pathway enrichment based on Mfuzz clustering. **(A)** Heatmap of KEGG pathway enrichment. The heatmap scale is based on Z scores ranging from −2 (blue) to 2 (red) with a midpoint of 0 (white); *p*-value < 0.05. **(B,C)** KEGG pathway-based enrichment analysis is divided into two subgraphs: cluster 1 and cluster 4 **(B)**, and cluster 3 **(C)**. Cluster 1, cluster 4, and cluster 3 are represented in red, blue, and green, respectively. **(D–F)** Characteristic KEGG pathways with cluster 3 and 4 proteins were obtained. Cluster 4 proteins are labeled in red on the oxidative phosphorylation (OXPHOS) map **(D)**. Cluster 3 proteins are marked in green on both the linoleic acid map **(E)** and the arachidonic acid map **(F)**.

We performed KEGG pathway enrichment analysis to predict the potential roles of altered proteins following chronic stress stimulation and ZBPYR administration (Figure 5A; Supplementary Table S5). Proteins related to mitochondria-specific mechanisms (i.e., the tricarboxylic acid (TCA) cycle, oxidative phosphorylation (OXPHOS), and synthesis and degradation of ketone bodies) and liver-specific mechanisms (i.e., alanine, aspartate, and glutamate metabolism; tryptophan metabolism; cysteine and methionine metabolism; and arginine biosynthesis) formed a specific focus in cluster 4 (Figures 5A,B). In the OXPHOS pathway, there were 16 DEPs (i.e., Ndufs8, Atp5mf, Atp5f1b, Atp5f1d, Tcirg1, Cox5a, Atp5f1a, Sdhb, Atp5me, Uqcrc2, Atp5po, Ndufa10, Ndufa5, Uqcrc1, Sdhd, and Sdha) observed (Figure 5D). In addition, cluster 1 contained DEPs related to the insulin signaling pathway (Figure 5B). Proteins related to ER-specific mechanisms (i.e., protein processing in ER) and liver-specific mechanisms (i.e., linoleic acid metabolism, arachidonic acid metabolism, drug metabolism, retinol metabolism, and steroid hormone biosynthesis) formed a specific focus in cluster 3 (Figures 5A,C). Most notably, we also observed that the end of the linoleic acid (LA) pathway overlapped with the arachidonic acid (AA) pathway (Figures 5E,F). There were five DEPs (i.e., Cyp3a18, Cyp3a2, Cyp2c11, Cyp3a9, and Cyp3a23/3a1) and two DEPs (i.e., Cyp2c11 and Cyp2b3) observed in the LA and AA pathways, respectively. We selected one insulin signaling pathway-related DEP in cluster 1, 16 OXPHOS-related DEPs in cluster 4, and six LA and AA-related DEPs in cluster 3 (Table 1). Among the 16 DEPs related to OXPHOS, they were all up-regulated in the model:control group (ratio >1.2) and had a downward trend (0.85 < ratio < 1.05) in the ZBPYR:model group. The six DEPs that were related to LA and AA were all downregulated in the model:control group (ratio < 0.83) and had an upward trend (1 < ratio < 1.2) in the ZBPYR:model.

**TABLE 1:** Characteristic proteins that are differentially expressed in the control, model, and ZBPYR groups.

Group	Gene	Protein names	Relative ratio	
			Model vs. Control	ZBPYR vs. Model
Insulin signaling pathway	Flot1	Flotillin-1	1.15	0.90
Oxidative phosphorylation	Ndufs8	Complex I-23kD	1.28	0.99
	Atp5mf	ATP synthase subunit f, mitochondrial	1.63	1.02
	Atp5f1b	ATP synthase subunit beta	1.37	0.94
	Atp5f1d	ATP synthase F1 subunit delta	1.58	0.98
	Tcirg1	V-type proton ATPase subunit a	1.26	0.96
	Cox5a	Cytochrome c oxidase subunit 5A, mitochondrial	1.78	0.89
	Atp5f1a	ATP synthase subunit alpha, mitochondrial	1.38	0.94
	Sdhb	Succinate dehydrogenase [ubiquinone] iron-sulfur subunit, mitochondria	1.50	0.92
	Atp5me	ATP synthase subunit e, mitochondrial	1.38	1.03
	Uqcrc2	Cytochrome b-c1 complex subunit 2, mitochondrial	1.21	1.00
	Atp5po	ATP synthase subunit O, mitochondrial	1.34	0.99
	Ndufa10	NADH dehydrogenase [ubiquinone] 1 alpha subcomplex subunit 10, mitochondrial	1.31	0.97
	Ndufa5	NADH dehydrogenase [ubiquinone] 1 alpha subcomplex subunit 5	1.25	0.95
	Uqcrc1	Cytochrome b-c1 complex subunit 1, mitochondrial	1.45	1.00
	Sdhd	Succinate dehydrogenase [ubiquinone] cytochrome b small subunit, mitochondrial	1.65	0.94
	Sdha	Succinate dehydrogenase [ubiquinone] flavoprotein subunit, mitochondrial	1.30	0.95
Linoleic acid metabolism	Cyp3a18	Unspecific monooxygenase	0.61	1.15
	Cyp3a2	Cytochrome P450 3A2	0.68	0.98
	Cyp2c11	Cytochrome P450 2C11	0.59	0.94
	Cyp3a9	Cytochrome P450 3A9	0.81	1.03
	Cyp3a23/3a1	Unspecific monooxygenase	0.80	1.11
Arachidonic acid metabolism	Cyp2c11	Cytochrome P450 2C11	0.59	0.94
	Cyp2b3	Cytochrome P450 2B3	0.80	1.07

### Protein domain enrichment analysis of differentially expressed proteins in liver mitochondria-associated endoplasmic reticulum membrane based on soft clustering

**FIGURE 6:**
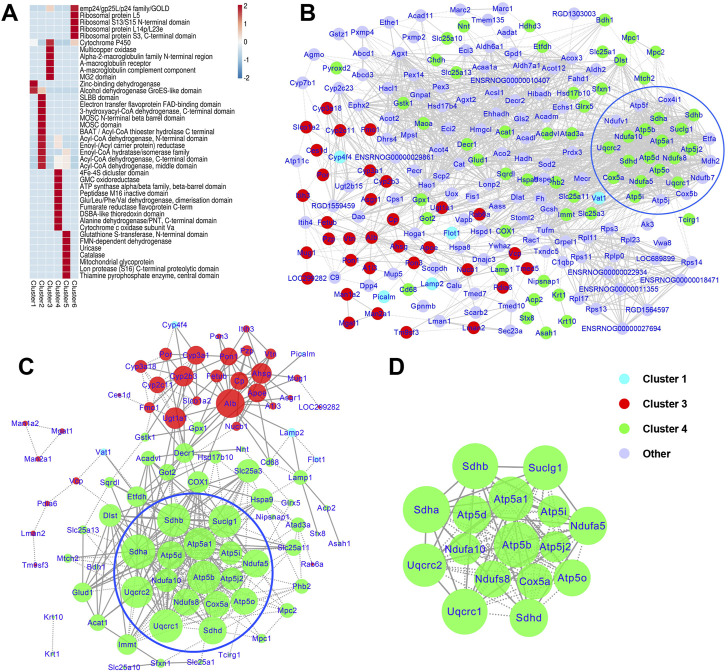
Protein domain enrichment analyses and protein-protein interaction (PPI) analysis of DEPs. **(A)** Protein domain enrichment analyses based on Mfuzz clustering. The heatmap scale is based on *Z* scores ranging from −2 (blue) to 2 (red) with a midpoint of 0 (white); *p*-value < 0.05. **(B)** PPI maps of the DEPs in the control, model, and ZBPYR groups. **(C,D)** PPI network and modules of cluster 1, 3, and 4 proteins. PPI **(C)** and modules **(D)** are proteins associated with the administration of T2DM under chronic psychological stress and ZBPYR treatment. The blue nodes belong to cluster 1, the red nodes belong to cluster 3, the green nodes be a part of cluster 4, and the purple nodes are the other DEPs. The sizes of the nodes reflect the node degree.

Domais are conserved parts of a given protein’s aminoacid sequence and structure, which can exist and function independently of the rest of the protein. Domain annotation and enrichment analysis were conducted to identify the domain characteristics of the DEPs altered by T2DM with chronic psychological stress and ZBPYR administration. Our results indicated that cytochrome P450, 4Fe-4S dicluster domain, and GMC oxidoreductase were highly enriched (Supplementary Table S6). Protein domains involved in the 4Fe-4S dicluster domain; GMC oxidoreductase; ATP synthase alpha/beta family, beta-barrel domain; peptidase M16 inactive domain; Glu/Leu/Phe/Val dehydrogenase, dimerization domain; fumarate reductase flavoprotein C-term; DSBA-like thioredoxin domain; alanine dehydrogenase/PNT, C-terminal domain; and cytochrome c oxid ase subunit Va were further identified to be highly enriched in cluster 4 (Figure 6A). The zinc-binding dehydrogenase and alcohol dehydrogenase GroES-like domains were significantly enriched in cluster 1. In cluster 3, various domains such as cytochrome P450 and multicopper oxidase were highly enriched. In short, these information suggest that proteins with multiple domain signatures were altered in response to T2DM with chronic psychological stress and ZBPYR administration.

### Protein-protein interaction network analysis of differentially expressed proteins in liver mitochondria-associated endoplasmic reticulum membrane based on soft clustering

Based on Mfuzz analysis, a total 234 DEPs were obtained. The PPI network contained 218 DEPs and their interactions (Figure 6B), of which Cat (cluster 5, degree = 62), Mdh2 (cluster 2, degree = 54), Atp5f1b (cluster 4, degree = 51), Atp5f1a (cluster 4, degree = 50), Sdha (cluster 4, degree = 49), and Suclg1 (cluster 4, degree = 49) were hub proteins that had high degrees of interaction. Among them, 24 proteins related to energy metabolism were evident (i.e., Atp5f1a, Atp5f1b, Atp5f1d, Atp5pb, Atp5me, Atp5pf, Atp5mf, Atp5po, Cox4i1, Cox5a, Cox5b, Etfa, Mdh2, Ndufa10, Ndufa5, Ndufb7, Ndufs8, Ndufv1, Sdha, Sdhb, Sdhd, Suclg1, Uqcrc1, and Uqcrc2). For further screening, the PPI network and modules of clusters 1, 3, and 4 were selected (Figure 6C). The top seven nodes with the highest degree in the key PPI were Sdha (degree = 27), Sdhb (degree = 26), Uqcrc1 (degree = 26), Atp5f1b (degree = 26), Suclg1 (degree = 25), Atp5f1a (degree = 25), and Uqcrc2 (degree = 24), all of which belonged to cluster 4. One significant functional module with the highest MCODE score (score = 15.733) was obtained from the PPI network (Figure 6D), and 16 DEPs were contained in the module, including one TCA cycle-related protein (Suclg1) and 15 mitochondrial respiratory chain-related DEPs (Ndufs8, Ndufa10 and Ndufa5 in Complex I; Sdha, Sdhb and Sdhd in Complex II; Uqcrc1 and Uqcrc2 in Complex III; Cox5a in Complex IV; and Atp5mf, Atp5f1b, Atp5f1d, Atp5f1a, Atp5me, and Atp5po in Complex V).

## Key differentially expressed proteins in liver mitochondria-associated endoplasmic reticulum membrane

**FIGURE 7:**
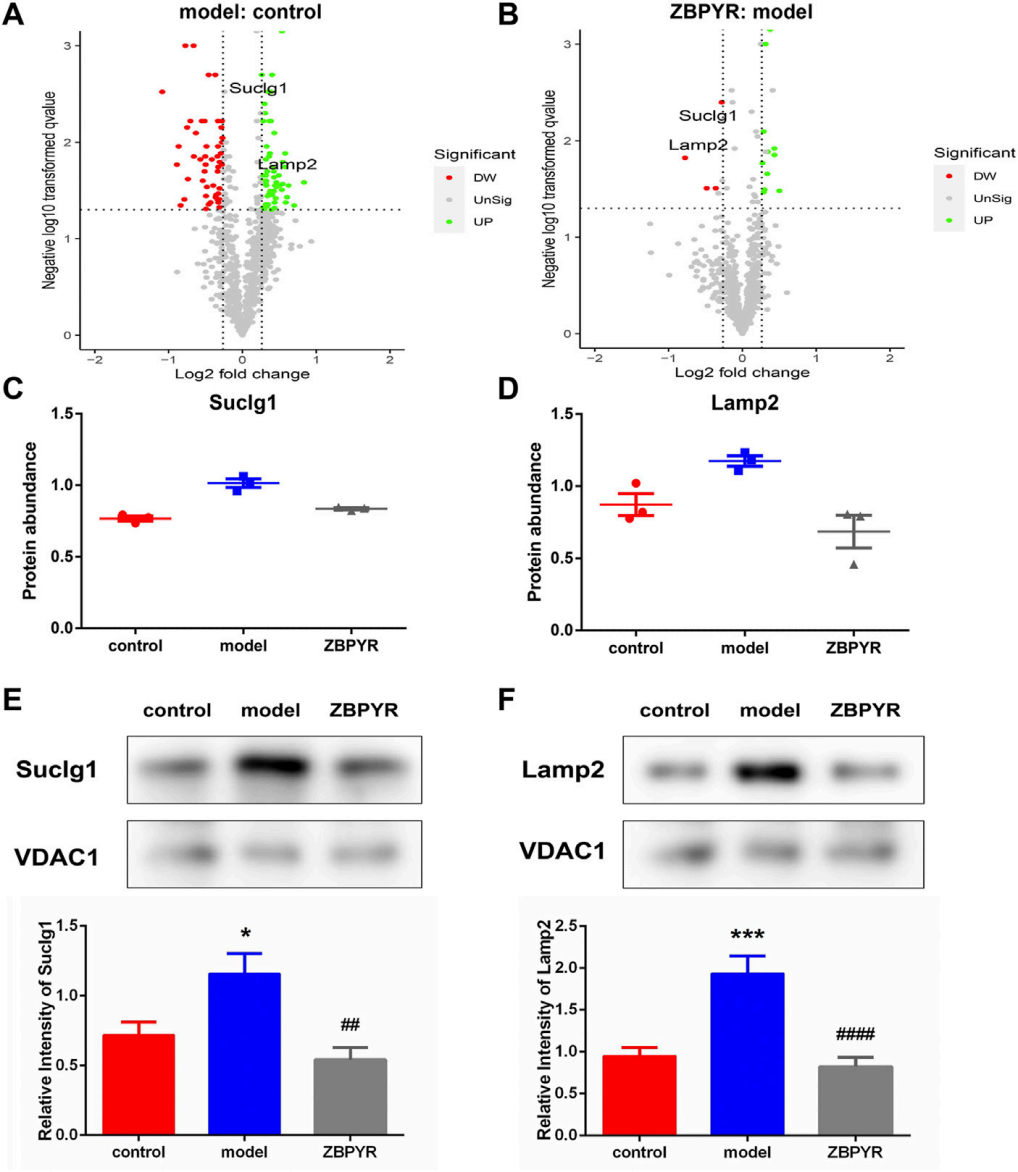
Screening of key proteins. **(A,B)** Volcano plots showing log2 fold-change (*x*-axis) and −log10 *p*-value (*y*-axis) for quantitative proteins in the model:control **(A)** and ZBPYR:model **(B)** groups. The decreased and increased proteins are marked by red and green, respectively. **(C,D)** Relative abundance of the two key liver MAM proteins in model:control and ZBPYR:model groups (mean ± SEM, *n* = 3 per group). Suclg1 **(C)** and Lamp2 **(D)** were consistent with the key protein screening criteria: 1) the fold change of protein relative abundance > ±1.2; 2) *t*-test *p*-value < 0.05; and 3) the opposite expression trend of upregulation and downregulation in the two groups. **(E,F)** Western blotting analysis for two key proteins. MAM fractions from the control, model, and ZBPYR groups were analyzed by Western blotting using antibodies against Suclg1 **(E)** and Lamp2 **(F)**. VDAC1 was used to ensure equal protein loading and transfer. The levels of Lamp2 and Suclg1 were normalized relative to VDAC1 levels. *p < 0.05, ***p < 0.001, ##*p* < 0.01, ####*p* < 0.0001 (mean ± SEM, *n* = 3 per group).

According to the key protein screening criteria and functional analysis, two common representative proteins in the model:control and ZBPYR:model groups were screened (Figures 7A,B). The results of proteomics showed that the expressions of Lamp2 and Suclg1 were significantly increased in the model:control group, and decreased in the ZBPYR:model group (Figures 7C,D). The immunoblotting patterns of these two proteins matched well with those selected in proteomic analysis (Figures 7E,F). Therefore, we have reason to believe that Lamp2 and Suclg1 may be influenced by T2DM with chronic psychological stress and by ZBPYR administration as underlying proteins.

## Discussion

There is increasing evidence indicates that MAM dysfunction is implicated in the pathogenesis of multiple diseases, including T2DM. MAM dysfunction mainly manifests as energy metabolism disorder, lipid synthesis and transport damage, mitochondrial dynamics disorders, abnormal autophagy, and disturbance of ER homeostasis (Rieusset, 2017; Cheng et al., 2020). By establishing the chronic psychological stress T2DM model, we attempted to elucidate the specific molecular mechanisms underlying T2DM with chronic psychological stress-related liver MAM dysfunction and to evaluate the effect of ZBPYR on T2DM with chronic psychological stress. Apart from obesity, hyperinsulinemia, hyperglycemia, and hyperlipidemia, ZDF rats have been reported to exhibit impaired spatial cognition (Kamal et al., 2013). Our previous research found that given chronic psychological stress (restriction, rotation, and congestion), ZDF rats exhibited persistent disruption of the hypothalamic-pituitary-adrenal axis, impaired exploratory-like behavior, and increased cognitive abnormalities, whereas ZBPYR treatment could significantly improve these symptoms (Xu et al., 2020). Additionally, elevations in stress-related indicators (corticosterone and adreno-cortico-tropic-hormone) may contribute to the subsequent hyperglycemia in ZDF rats.

Under chronic psychological stress, T2DM rats’ weight, food intake, water consumption, and blood glucose were significantly increased, and insulin sensitivity and exploratory behavior were decreased. ZBPYR had little effect on body weight, but improved other conditions. Proteomics analysis of liver MAM fractions revealed that T2DM with chronic psychological stress-related symptoms and behavioral changes were in accordance with differential expression of 234 proteins. These proteins were related to TCA cycle, energy metabolism, amino acid metabolism, lysosome-related processes, lipid metabolism (i.e., linoleic acid and arachidonic acid metabolism), and the insulin signaling pathway. Among them, the expression levels of TCA cycle-related protein Suclg1 and lysosome-related protein Lamp2 were both significantly upregulated in the model:control group and reduced in the ZBPYR:model group, indicating that liver MAM in T2DM rat with chronic psychological stress suffers from dysfunctions of carbohydrate metabolism and lysosome-related processes.

### Energy metabolism

Numerous researches have demonstrated that the pathogenesis of T2DM may accompany abnormal energy metabolism. Indeed, we found a large number of MAM proteins involved in energy metabolism in the liver. Forty-six DEPs in liver MAM were involved in the mitochondrial respiratory chain, including: Ndufs3, Ndufab1, Ndufa10l1, Ndufb5, ND5, Ndufb6, ND2, Ndufv2, Ndufa5, Ndufs1, Ndufa9, Ndufv1, Ndufs8, Ndufb7, and Mt-nd4 in Complex I; Sdhd, Sdha, Sdhc, and Sdhb in Complex II;Uqcrc2, Uqcrc1, and Uqcrfs1 in Complex III; Cox4i1, COX2, Cox5b, Cox5a, Cox1, and Cox7c in Complex IV, and Atp5f1a, Atp5f1, Atp5h, Atp5f1b, Atp5c1, Atp5mf, Atp5po, Atp5j, Atp5f1d, Atp5me, Atp5l, and Tcirg1 in Complex V. In particular, the 16 DEPs related to the mitochondrial respiratory chain belonged to cluster 4, including: Ndufs8, Atp5mf, Atp5f1b, Atp5f1d, Tcirg1, Cox5a, Atp5f1a, Sdhb, Atp5me, Uqcrc2, Atp5po, Ndufa10, Ndufa5, Uqcrc1, Sdhd, and Sdha. Interestingly, the DEPs related to oxidative phosphorylation in cluster 4 were upregulated in the model:control group. It can be seen that the changes of protein in T2DM are closely associated with the regulation of mitochondrial function and oxidative stress. *β*-Cells sense the fluctuations of plasma glucose levels and secrete insulin accordingly, and mitochondria play an important role in the regulation of insulin secretion. Therefore, increased expression of mitochondrial respiratory chain complex subunits exposed to T2DM may be an adaptive response to the long-term sustained increase in blood glucose levels in the liver. T2DM simultaneously increased the expression of oxidative phosphorylation-related proteins and antioxidant enzymes (such as Sod2) in the liver, suggesting that T2DM exposure changes the organism’s redox balance and triggers oxidative stress. Several studies have documented changes in protein levels associated with oxidative phosphorylation and mitochondrial dysfunction in the liver of diabetic monkeys and a high fat diet/streptozotocin-induced T2DM mouse model (Wang et al., 2018a; Zhu et al., 2019). In general, the disturbance of mitochondrial energy metabolism-associated proteins responsible for ATP generation through oxidative phosphorylation plays an important, and possibly central, role in the development of T2DM with chronic psychological stress.

### Tricarboxylic acid cycle

The TCA cycle is the central hub for mitochondrial energy production, coordinating glucose, fatty acid and amino acid metabolism. T2DM affects TCA cycle metabolism in various tissues (such as the liver). In this study, T2DM caused the increase of several TCA cycle-related DEPs (such as Suclg1) in cluster 4. Succinyl-CoA ligase subunit alpha (Suclg1) takes mitochondria as the target and catalyzes the conversion of Succinate-CoA and ADP or GDP to succinate and ATP or GTP. Using iTRAQ-MALDI-TOF/TOF technology, a previous study showed that Suclg1 is upregulated in a diabetic mouse model at week 15 (Mukul et al., 2012). Therefore, it is natural to suppose that abnormal TCA cycle may be a feature of MAM dysfunction in the progression of T2DM with chronic psychological stress.

### Amino acid metabolism

Disorders of amino acid metabolism are an important molecular mechanism of T2DM. A long-term, high glucose environment will inevitably lead to disorders of amino acid metabolism and destroy the steady-state of the glutamate-glutamine cycle. In this study, T2DM with chronic psychological stress caused the increase of several amino acid metabolism-related DEPs (such as Got2, Acat1, and Hsd17b10) in cluster 4. Glutamic-oxaloacetic transaminase 2 (Got2, a.k.a., aspartate aminotransferase 2) is a pyridoxal phosphate-dependent enzyme present in the inner mitochondrial membrane. As a member of the malate-aspartate shuttle, it plays a prominent part in the intracellular NAD(H) redox homeostasis. In addition, Got2 is important for metabolite exchange between mitochondria and cytosol, as well as for amino acid metabolism. Aspartate transaminase levels and activity were significantly increased in the diabetic group compared to the normal group (Ma et al., 2020; Wang et al., 2013). Acetyl-CoA acetyltransferase 1 (Acat1) mediates the reversible conversion of two acetyl-CoA molecules to acetoacetyl-CoA. It is demonstrated that the proportion of ACAT1 in the MAM is higher than that of endoplasmic reticulum and mitochondria (Haapalainen et al., 2007). This enzyme catalyzes the final step in the branched-chain amino acid and fatty acid degradation pathways, where the acetyl-CoA produced is used as an input for the TCA cycle. When the energetics is conducive to product acetoacetyl-CoA, the metabolite is used for ketone body synthesis (Haapalainen et al., 2007). Therefore, Acat1 is functionally placed at the intersection of cellular pathways in response to cellular energy status. Hydroxysteroid 17-beta dehydrogenase 10 (Hsd17b10), as an NAD^+^-dependent dehydrogenase, has a wide range of substrates and functions, including but not limited to the metabolism of branched-chain fatty acids, the catabolism of isoleucine (Zschocke et al., 2000), and activation or inactivation of potent sex hormones or steroid modulators (He et al., 2005). In addition, its non-enzymatic activitity seems to be of vital importance in mitochondrial health. In conclusion, using these amino acids as early potential biomarkers of T2DM with chronic psychological stress may provide new insights into the early pathological changes and molecular mechanisms of diabetes mellitus.

### Lysosome-related processes

The MAM-mediated mitochondrial-ER connection is dynamic and reversible, involving the recruitment and depolymerization of various proteins. “MAM enriched proteins” refer to those proteins that are mainly present in the MAM but also distributed in other organelles such as the lysosom. Interestingly, we found that three DEPs (Lamp1, CD68, and Asah1) in cluster 4 and one key protein (Lamp2) in cluster 1 were involved in lysosomal dysfunction in this study. Lysosome-associated membrane glycoprotein 1 and 2 (Lamp1 and Lamp2) are both members of the membrane glycoprotein family, which are necessary for the proper fusion of lysosomes with autophagosomes at the late stage of autophagy (Giansanti et al., 2011). The autophagy-lysosome pathway is involved in protein and organelle degradation. Lamp1 and Lamp2, markers of lysosome function, are reduced in the hippocampus of diabetic rats (Ma et al., 2017b). These researches suggest that diabetes mellitus activates autophagy, but impairs lysosome function. CD68 Molecule (CD68), as a member of the lysosomal/endosomal-related membrane glycoprotein family, is considered to be a pan-macrophage marker, which is mainly expressed on the intracellular lysosomes of tissue macrophages/monocytes, such as microglia and Kupffer cells. Immune cells such as macrophages may be another source of islet inflammation and take part in the development of T2DM (Weaver et al., 2020). An increase in the number of islet-associated CD68^+^ macrophages are observed in T2DM patients and T2DM animal models, such as Goto-Kakizaki rats and db/db mice (Ehses et al., 2007). Acid ceramidase (Asah1), present in the lysosomal compartment, as a ceramidase, is a key enzyme in maintaining ceramide/sphingosine homeostasis in cells, and is also a key regulator of signals that tilt the balance between cell survival and death (Duarte et al., 2020). Under the condition of metabolic disorders, the activity of acid ceramidase or the expression level of Asah1 in type 2 diabetes is changed (Chavez et al., 2005). According to other reports, Asah1 may not be involved in *β*-Cell function but in insulin signaling (Chavez et al., 2005; Bruce et al., 2006). In summary, the complexity, versatility, and dynamics of MAM protein composition creates conditions for MAM to participate in the intervention of T2DM with chronic psychological stress and ZBPYR treatment, which is accompanied by changes in lysosome-related proteins.

### Lipid metabolism

Abnormal lipid metabolism exists in the clinical pathogenesis of T2DM. MAM is enriched with lipid-metabolizing enzymes and mediates lipid transport between the ER and mitochondria, which makes MAM play an critical role in maintaining normal lipid metabolism. In the current study, T2DM with chronic psychological stress caused a simultaneous decrease of Cyp3a18, Cyp3a2, Cyp2c11, Cyp3a9, Cyp3a73, and Cyp2b3 in cluster 3. Among them, Cyp3a18, Cyp3a2, Cyp2c11, Cyp3a9, and Cyp3a73 are involved in linoleic acid metabolism, and Cyp2b3 and Cyp2c11 are involved in arachidonic acid metabolism. Cytochromes P450 (CYP) are a group of heme-thiolate monooxygenases that take part in the metabolism of steroid hormones and fatty acids. Both Cyp3a2 and Cyp2c11 are members of the cytochrome P450 family. Cyp3a2, a male-specific CYP, is predominantly expressed in the livers of adult male rats. Cyp2c11 is considered to be the major CYP in rats, accounting for 54% of the total CYP content. Through clinical trials and animal experiments, the expression and activity of CYP are proverd to be significantly changed by diabetes mellitus (Hu et al., 2011; Kataoka et al., 2005). Studies have shown that CYP expression and activity are altered in diabetic conditions depending on the type of diabetes and CYP subtypes. For example, hepatic Cyp2c11 expression is not significantly different in non-obese type 2 diabetic Goto-Kakizaki rats (Oh et al., 2012), but is reduced in five-week-old ZDF rats (Park et al., 2016), streptozotocin-induced diabetic rats (Ahn et al., 2010) and high-fat diet-fed and low-dose streptozotocin-induced diabetic rats (Vornoli et al., 2014). Nevertheless, there are some inconsistencies regarding Cyp3a2 expression in the existing literature; five-week-old ZDF rats have decreased Cyp3a2 levels in the liver (Park et al., 2016), but Goto-Kakizaki rats (Oh et al., 2012) and high-fat diet-fed and low-dose streptozotocin-induced diabetic rats have increased Cyp3a2 levels (Chen et al., 2011). In conclusion, it is reasonable to believe that abnormal lipid metabolism in liver MAM may be common in chronic psychological stress T2DM.

### Insulin signaling pathway

A large number of studies have confirmed that insulin resistance and insulin signal transduction defects are important factors in T2DM with chronic psychological stress that can inhibit the PI3K/Akt signaling pathway and affect physiological processes such as autophagy and inflammation. In this study, flotillin-1 in cluster 1 involving the insulin signaling pathway was discovered to be a DEP. Flotillins, as hydrophobic proteins, reside in lipid rafts of intra- and extracellular vesicles. There are two flotillin paralogues, termed as flotillin-1 and flotillin-2. Flotillins (mainly flotillin-1) have been proved to be abundantly expressed in pyramidal neurons of the cortex, as well as in the astrocytes of the white matter of normal human brain tissue (Kokubo et al., 2000). Livers of obese, T2DM KKA^y^ mice have 60%–70% less flotillin-1 mRNA and protein than that of non-diabetic KK livers (Chen et al., 2018). Taken together, these findings suggest that Flot1 may be an intervention target for T2DM with chronic psychological stress and ZBPYR treatment.

## Conclusion

**FIGURE 8:**
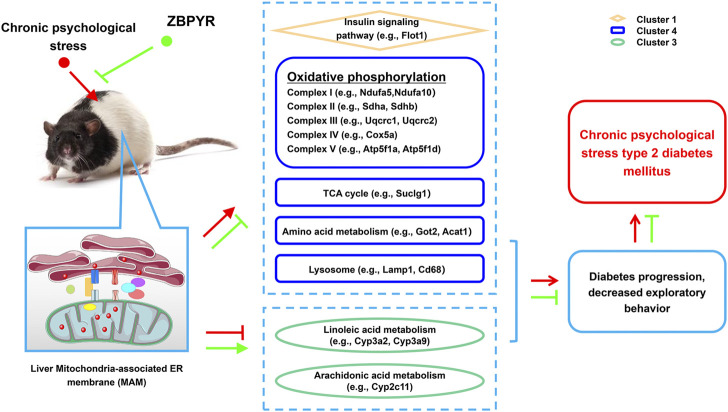
Potential mechanism of T2DM with chronic psychological stress and ZBPYR treatment. Compared with control rats, the proteomics of liver MAM in model rats changed, including insulin signaling pathway, oxidative phosphorylation, TCA cycle, amino acid metabolism, lysosome, linoleic acid metabolism, and arachidonic acid metabolism-related proteins, while ZBPYR treatment caused proteomic changes in liver MAM in ZBPYR rats by regulating the expression of the above proteins. This may underlie the molecular basis of T2DM with chronic psychological stress and the potential therapeutic mechanism of ZBPYR.

In conclusion, there are various proteins differentially expressed in liver MAM of ZDF rats that were exposed to chronic psychological stress, which exhibited aggravated diabetes symptoms and reduced exploratory behavior (Figure 8). These proteins included ETC-related proteins (Ndufs8, Atp5mf, Atp5f1b, Atp5f1d, Tcirg1, Cox5a, Atp5f1a, Sdhb, Atp5me, Uqcrc2, Atp5po, Ndufa10, Ndufa5, Uqcrc1, Sdhd, and Sdha), TCA cycle-related proteins (Suclg1), amino acid metabolism-related proteins (Got2, Acat1, and Hsd17b10), lysosome-related proteins (Lamp1, Cd68, Asah1, and Lamp2), linoleic acid metabolism-related proteins (i.e., Cyp3a2, Cyp2c11, and Cyp3a9), arachidonic acid metabolism-related proteins (Cyp2c11), and an insulin signaling-related protein (Flot1). The expressions of two key proteins (Lamp2 and Suclg1) were notably increased in the model:control group and decreased in the ZBPYR:model group. Taken together, our data suggest that interventions that regulate MAM proteins, especially Lamp2 and Suclg1, in the liver may provide new opportunities for the treatment of T2DM with chronic psychological stress.

## Data Availability

The data presented in the study are deposited in the ProteomeXchange repository, accession number PXD029226.

## Supplementary material

SUPPLEMENTARY FIGURE S1 Schematic diagram of MAM isolation and purity confirmation of the MAM fraction by WB. MAM was isolated from rat livers by differential centrifugation and self-forming Percoll gradient centrifugation. Other cell organelles, such as crude mitochondria and ER were obtained by multiple centrifuge steps. **(B)** Western blot analysis of organelle markers in isolated MAM from the liver were enriched for FACL4, and were free from tubulin and COXIV contamination. Mito, mitochondria; ER, endoplasmic reticulum; MAM, mitochondria-associated ER membrane; Cyto, cytosol.

SUPPLEMENTARY FIGURE S2 Differential protein expression overview and Mfuzz clustering. Each trace is color-coded in terms of the membership value of respective cluster. Protein expression by Mfuzz clustering is in accordance with the protein expression patterns in each group. (Cluster 1, *n* = 5; Cluster 2, *n* = 68; Cluster 3, *n* = 44; Cluster 4, *n* = 57; Cluster 5, *n* = 28; Cluster 6, *n* = 32).

## Abbreviations

AA, arachidonic acid; Acat1, acetyl-CoA acetyltransferase 1; ACSL4/FACL4, acyl-CoA synthase long chain 4; Asah1, acid ceramidase; BP, biological process; CC, cellular component; CYP, cytochromes P450; DEPs, differentially expressed proteins; ER, endoplasmic reticulum; GO, gene ontology; Got2, glutamic-oxaloacetic transaminase 2; Hsd17b10, hydroxysteroid 17-beta dehydrogenase 10; iTRAQ, isobaric tags for relative and absolute quantitation; KEGG, kyoto encyclopedia of genes and genomes; LA, linoleic acid; Lamp1, lysosome-associated membrane glycoprotein 1; Lamp2, lysosome-associated membrane glycoprotein 2; LC-MS/MS, liquid chemistry-mass spectrometry/mass spectrometry; MAM, mitochondria-associated endoplasmic reticulum membrane; MF, molecular function; OXPHOS, oxidative phosphorylation; RBG, random blood glucose; SEM, standard error of measurement; Suclg1, succinyl-CoA ligase subunit alpha; T2DM, type 2 diabetes mellitus; TCA, tricarboxylic acid; ZBPYR, ZiBuPiYin recipe; ZDF, Zucker diabetic fatty; ZL, Zucker lean.
