# Supplementary figures and images for: Liver mitochondria-associated endoplasmic reticulum membrane proteomics for studying the effects of ZiBuPiYin recipe on Zucker diabetic fatty rats after chronic psychological stress
Source: Front Cell Dev Biol. 2022 Nov 3;10:995732. doi: 10.3389/fcell.2022.995732 (PMC9669571; doi:10.3389/fcell.2022.995732)

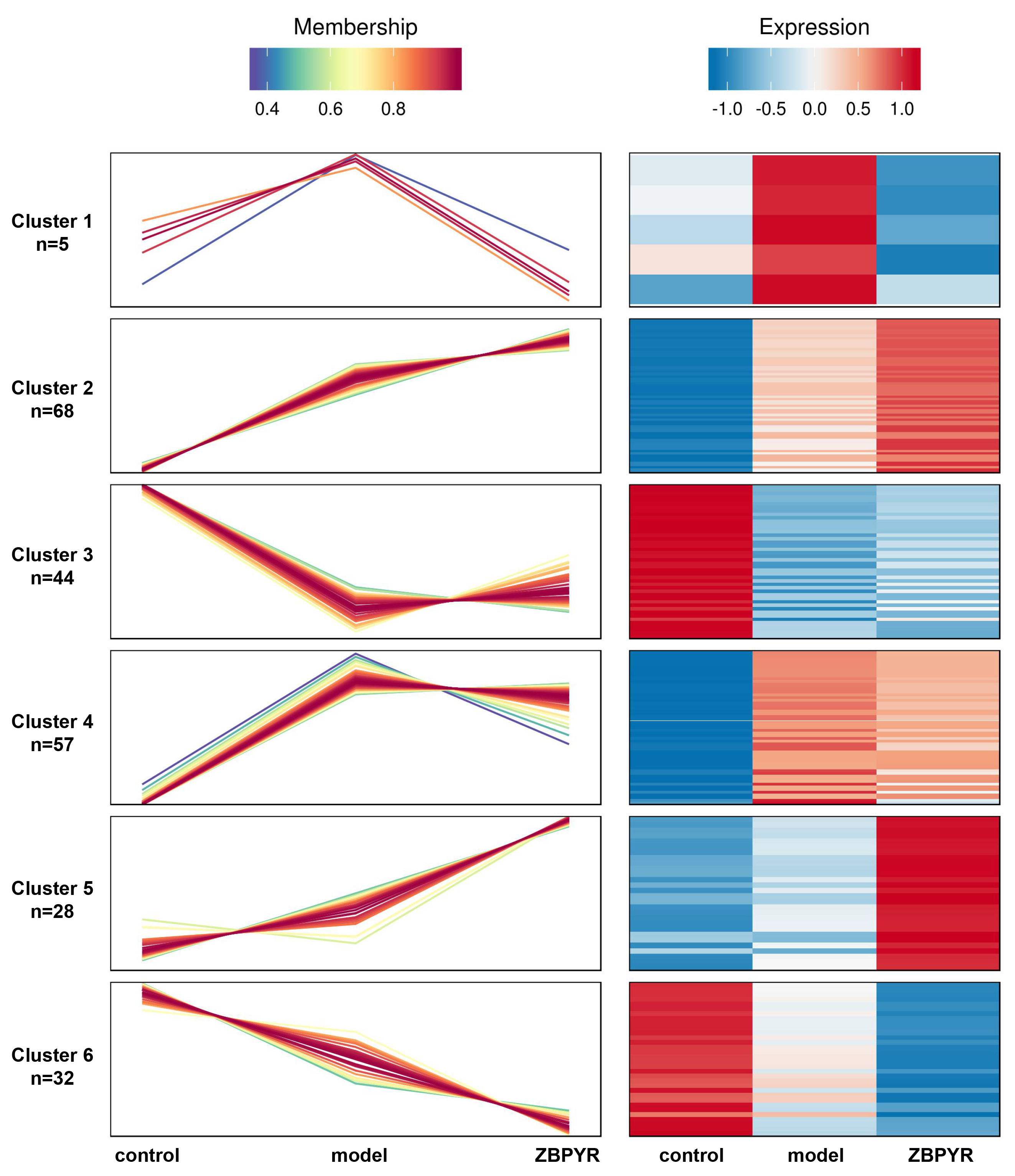

Supplement: Supplementary file 1 [file DataSheet1.zip › Data Sheet 1/221013 Supplementary materials/Figure S2.tif]
